# Association Between Autophagy and Neurodegenerative Diseases

**DOI:** 10.3389/fnins.2018.00255

**Published:** 2018-05-22

**Authors:** Nobuhiro Fujikake, Minkyoung Shin, Shigeomi Shimizu

**Affiliations:** Department of Pathological Cell Biology, Medical Research Institute, Tokyo Medical and Dental University, Tokyo, Japan

**Keywords:** autophagy, neurodegenerative disease, Alzheimer disease, tauopathy, Parkinson disease, amyotrophic lateral sclerosis, polyglutamine disease

## Abstract

Autophagy is a phylogenetically conserved mechanism that controls the degradation of subcellular constituents, including misfolded proteins, and damaged organelles. The progression of many neurodegenerative diseases is thought to be driven by the aggregation of misfolded proteins; therefore, autophagic activity is thought to affect disease severity to some extent. In some neurodegenerative diseases, the suppression of autophagic activity accelerates disease progression. Given that the induction of autophagy can potentially mitigate disease severity, various autophagy-inducing compounds have been developed and their efficacy has been evaluated in several rodent models of neurodegenerative diseases.

## Introduction

A common pathology shared by several neurodegenerative diseases, including Alzheimer disease (AD), Parkinson disease (PD), amyotrophic lateral sclerosis (ALS), and the polyglutamine (polyQ) diseases is the accumulation of misfolded proteins. Because autophagy is a cellular function that degrades abnormal proteins, including those that are misfolded, the onset and progression of these diseases are affected by autophagic activity within neurons. In this review, we first summarize the molecular mechanism and physiological relevance of autophagy. Next, we review how autophagy is involved in the pathology of several neurodegenerative diseases. We also summarize the therapeutic effects of autophagy-modulating compounds on these neurodegenerative diseases.

## Molecular mechanisms of autophagy

Autophagy is a catabolic process in which cellular contents, including proteins, lipids, and entire organelles, are degraded by lysosomal lytic enzymes. Autophagy constitutively functions at low levels but is highly induced by a variety of cellular stressors, such as nutrient starvation, growth factor withdrawal, DNA damage, the accumulation of abnormal proteins, and organelle damage. In many physiological and pathological contexts, autophagy protects cells by facilitating the degradation of superfluous or damaged cellular constituents, for the subsequent recycling of amino acids, lipids, nutrients, and metabolites.

There are at least three distinct autophagic mechanisms, namely, macroautophagy, microautophagy, and chaperone-mediated autophagy. Macroautophagy is believed to be the core pathway for degrading cytoplasmic proteins and organelles (Nakatogawa et al., 2009). In this process, cytoplasmic components and organelles are incorporated into double-membrane structures called autophagosomes. Subsequently, the outer membrane of the cellular constituents are broken down by acid hydrolases (Mizushima et al., 2002). The term “autophagy” is usually used synonymously with macroautophagy (hereafter referred to simply as “autophagy” unless otherwise indicated). The second type of autophagy is microautophagy, which occurs via direct invagination of the lysosomal membrane to engulf cellular constituents, followed by closure of the membrane pocket and degradation of the constituents within the lysososomal lumen (Li et al., 2012). Microautophagy can deliver entire organelles, such as peroxisomes, directly into lysosomes. The third type of autophagy is chaperone-mediated autophagy. During chaperone-mediated autophagy, cytoplasmic proteins with a targeting motif interact with the cytosolic heat shock cognate 70 chaperone, which delivers the proteins to the lysosomal membranes (Kaushik and Cuervo, 2012). Substrate proteins are subsequently recognized by the lysosomal receptor and are unfolded to penetrate the lysosomal membrane through a multimeric complex. After substrate translocation, rapid degradation of substrate proteins occurs in the lysosomal lumen. Thus, chaperone-mediated autophagy enables targeted protein degradation as opposed to the nonspecific or “bulk” protein degradation that occurs during macroautophagy.

**Figure 1:**
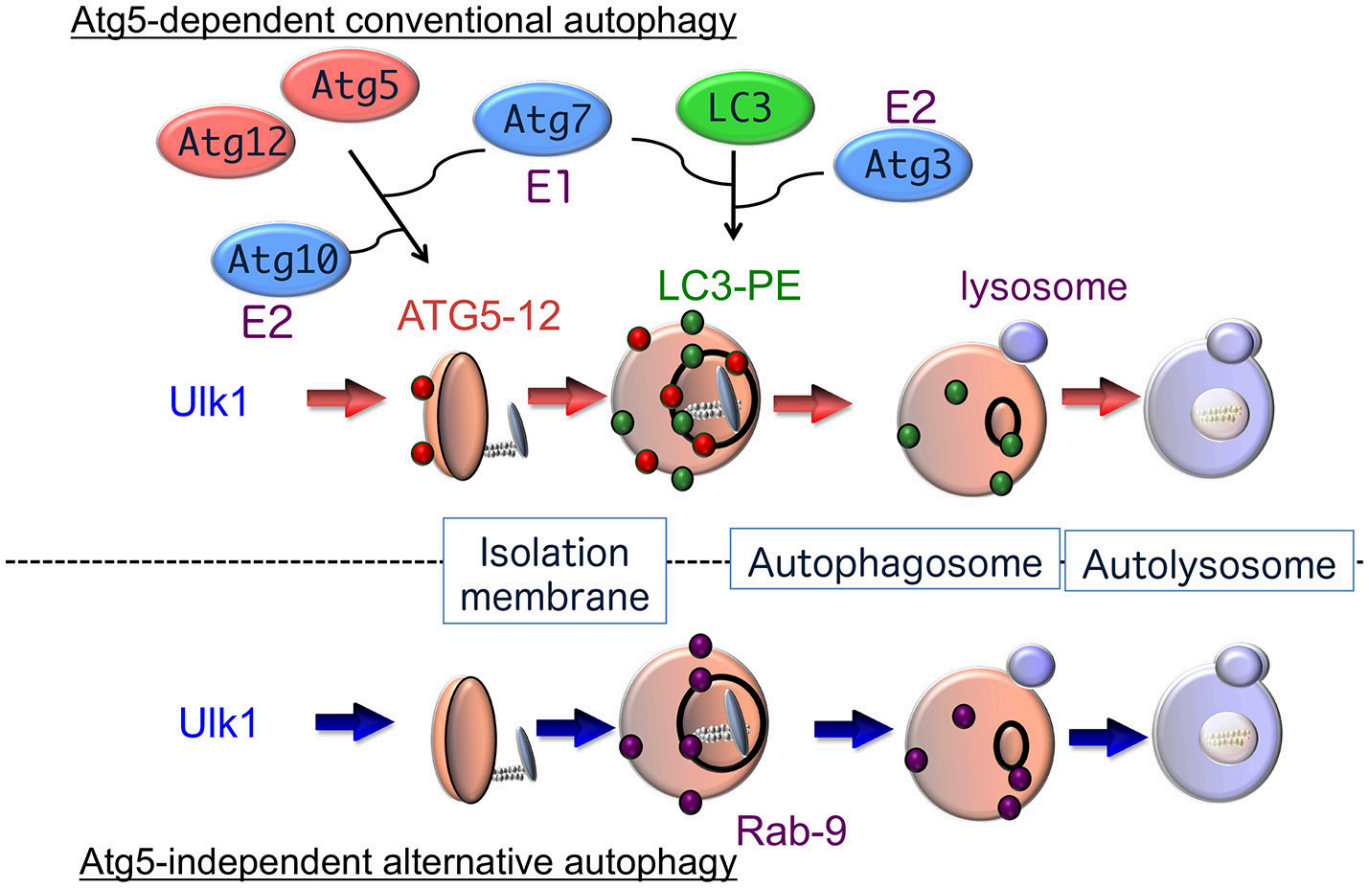
Hypothetical model of autophagy. There are at least two modes of macroautophagy, i.e., conventional and alternative autophagy. Conventional autophagy requires Atg5 and Atg7, is associated with LC3 modification, and is thought to originate from the ER membrane. After Ulk1 stimulation, Atg5-12 complexes (red circles) attach to isolation membranes, and then LC3-PE puncta (green circles) are incorporated into autophagosomal membranes. In contrast, alternative autophagy occurs independently of Atg5 and Atg7, as well as LC3 modification. The generation of autophagic vacuoles in alternative autophagy is mediated by the fusion of isolation membranes with vesicles derived from the *trans*-Golgi as well as late endosomes, in a Rab9-dependent manner. After Ulk1 stimulation, Rab9 puncta (purple circles) attach to autophagic membranes.

Analysis of the molecular mechanisms involved in autophagy began with a genetic approach using autophagy-defective mutant yeast strains (Nakatogawa et al., 2009). Subsequently, many mammalian homologs of yeast autophagy proteins and their biochemical functions have been identified (Figure 1). It is now accepted that autophagy is driven by more than 30 autophagy-related proteins (Atgs) (Mizushima and Komatsu, 2011). Atg1 was the first such protein identified and shown to have intrinsic serine/threonine kinase activity, which is essential for the initiation of autophagy (Kabeya et al., 2005). Autophagy is regulated by phosphatidylinositol 3-kinase (PI3K) type I and type III. PI3K type I is activated by growth factors, and suppresses autophagy through the regulation of mammalian target of rapamycin (mTOR). Conversely, PI3K type III, which exists in a multiprotein complex including Atg6 (also called Beclin 1), facilitates the generation of the omegasomes, which is the initial membrane component of the isolation membrane (Axe et al., 2008). Isolation membranes subsequently expand, curve and close by two conjugation pathways, namely, the Atg5–Atg12 pathway and the microtubule-associated protein 1 light chain 3 (LC3) pathway (Mizushima and Komatsu, 2011). The ubiquitin-like conjugation of phosphatidylethanolamine (PE) to LC3 couples with the translocation of LC3 from the soluble fraction to autophagic membranes. Therefore, the localization of LC3-PE in autophagic membranes is a reliable marker of autophagy. The following lines of evidence indicate that the Atg5–Atg12 pathway is essential for the initiation of autophagy: (1) yeast Atg5 is crucial for autophagy, (2) autophagy is suppressed in some cell types from Atg5-deficient mice, and (3) LC3-PE formation has never been detected in Atg5-deficient cells. However, Atg5-deficient mouse embryos develop normally until the perinatal period (Kuma et al., 2004; Komatsu et al., 2005), suggesting that an alternative autophagic pathway may exist that compensates for the lack of Atg5-dependent autophagy in embryonic mutant mice.

In fact, we found that treatment with etoposide, a topoisomerase inhibitor that induces DNA strand breaks in mitotic cells, causes the formation of autophagic structures even in Atg5-deficient mouse embryonic fibroblasts (MEFs). The numbers and sizes of these autophagic vacuoles were equivalent in wild-type and Atg5-deficient MEFs, and the morphology of these etoposide-induced autophagic structures was indistinguishable from the autophagic vacuoles observed during starvation-induced autophagy (Nishida et al., 2009). Therefore, MEFs appear to perform two distinct types of autophagy, an Atg5-dependent conventional type and an Atg5-independent alternative type, which show similar morphological characteristics (Figure 1). Unlike Atg5-dependent autophagy, the molecules required for and the physiological roles of alternative autophagy have not yet been elucidated.

## The Ulk1 complex

Molecular mechanisms of conventional autophagy have been extensively studied using nutrient-starved cells, by referring to the information obtained from starved yeast cells, in which functional complexes containing Atg proteins drive for the formation of autophagic vacuoles. Starvation-induced autophagy begins from modifications in mTor and AMK-activated protein kinase (AMPK), which are well-established cellular nutritional sensors. mTor is a member of the serine/threonine protein kinase family and serves as a core component of two distinct protein complexes, mTor complex 1 (mTorc1) and mTorc2, which regulate different cellular processes. In healthy cells, mTorc1 suppresses autophagy by phosphorylating and inactivating Unc-51-like kinase 1 (Ulk1), a homolog of yeast Atg1 and an initiator of mammalian autophagy. AMPK is another kinase that plays a role in cellular energy homeostasis, and also suppresses autophagy by phosphorylating different amino acid residues of Ulk1. Upon nutrient starvation, Ulk1 is dephosphorylated by protein phosphatase 2A (PP2A) and translocates to limited membranes together with Fip200, Atg13, and Atg101. The membrane-associated ULK1 complex then phosphorylates multiple substrates, leading to the induction of conventional autophagy.

The Ulk1 protein is also crucial for alternative autophagy. Addition of etoposide induced the accumulation and autophosphorylation of Ulk1 in Atg5-deficient MEFs. Furthermore, no autophagic membranes were observed within Ulk1-silenced Atg5-deficient MEFs in response to etoposide. Similar results were also observed when Fip200, another component of the Ulk1 complex, was silenced. Thus, Ulk1 functions in the initiation of both conventional and alternative autophagy (Figure 1) (Nishida et al., 2009). The mechanism involved in the specific activation of each pathway by different stressors remains unclear. These two forms of autophagy may be selectively activated by the phosphorylation and activation of different Ulk1 substrates.

## The PI3K complex

The class III PI3 kinase Vps34 phosphorylates PI at the 3′-hydroxyl position to produce PI3P, an abundant component of autophagosomal membranes that is essential for autophagosome formation (Kihara et al., 2001). An autophagosome formation-specific PI3 kinase complex composed of Atg14L, Beclin 1, Vps15, and Vps34 was identified (Itakura et al., 2008). Two additional Vps34 complexes, Vps34-Vps15-Beclin 1-UVRAG and Vps34-Vps15-Beclin 1-UVRAG-Rubicon, function in the endocytic pathway and in the regulation of autophagosome/lysosome fusion, respectively (Matsunaga et al., 2009) (Zhong et al., 2009). Beclin 1 is also regulated by B-cell leukemia/lymphoma-2 (Bcl-2) family proteins, such as Bcl-2, Bcl-x_L_, and Bim (Levine et al., 2008). The protein double FYVE domain-containing protein 1 has been identified as a downstream effector of PI3P and it is concentrated within membrane domains of the endoplasmic reticulum (ER) that are sites of isolation membrane creation (Axe et al., 2008). The ULK1 complex was also reported to associate with the autophagosome formation-specific PI3K complex to generate isolation membranes. This PI3K complex is also crucial for alternative autophagy, as silencing Vps34 and Beclin 1 inhibited the alternative pathway (Nishida et al., 2009).

## Atg5-Atg12 and LC3 pathways

Two unique ubiquitin-like conjugation systems, the Atg5–Atg12 pathway and the LC3 pathway, are believed to be essential for the induction of conventional autophagy (Figure 1). The Atg5–Atg12 pathway is initiated when Atg12 is conjugated to Atg5 by Atg7 (E1 enzyme) and Atg10 (E2 enzyme) (Mizushima and Komatsu, 2011), and the Atg5–Atg12 conjugate further binds with Atg16 to generate an Atg5–Atg12/Atg16 complex. The second protein conjugation system is controlled by the Atg8 homolog LC3, gamma-aminobutyric acid receptor-associated protein (GABARAP), and Golgi-associated ATPase enhancer of 16 kD (GATE-16). During autophagy, these proteins are cleaved by the cysteine protease Atg4, resulting in the exposure of a glycine residue, which is then catalyzed by Atg7 (E1 enzyme), Atg3 (E2 enzyme), and the Atg5–Atg12/Atg16 complex (E3 enzyme). PE then binds to the exposed glycine residue and acts to localize these proteins to isolation membranes and autophagosome membranes. The PE-conjugated forms of LC3, GABARAP, and GATE-16 (termed LC3-II, type II GABARAP, and type II GATE16) are reliable markers of conventional autophagy (Kabeya et al., 2000).

Although Ulk1 and PI3K complexes participate in both conventional and alternative autophagy, neither the Atg5–Atg12 nor the LC3 conjugation pathway is required for alternative autophagy. Furthermore, the conversion of LC3-I to PE-conjugated LC3-II does not occur in alternative autophagy (Nishida et al., 2009). Thus, it is unknown as to how extension and closure of autophagic membranes are accomplished during alternative autophagy without these two ubiquitin-like systems. Detailed morphological analysis has, however, provided some clues. We demonstrated that elongation and closure of isolation membranes, presumably derived from trans-Golgi membranes are generated by fusion with endosomal vesicular membranes (Nishida et al., 2009). The involvement of trans-Golgi/endosomal fusion in the extension and closure of isolation membranes was confirmed by the colocalization of mannose-6-phosphate receptors (a trans-Golgi/late endosomal marker) and syntaxin 7 (a late endosomal marker) with Lamp2-positive vacuoles in etoposide-treated Atg5-deficient MEFs (Nishida et al., 2009). The formation of isolation membranes by trans-Golgi/endosomal fusion is also supported by studies showing a requirement for Rab9, a GTPase required for the trafficking of proteins from late endosomes to trans-Golgi membranes (Kabeya et al., 2000). First, GFP-Rab9 was colocalized with autolysosomes in etoposide-treated Atg5-deficient MEFs, and this colocalization was increased by the expression of GFP-Rab9^Q66L^, a constitutively active Rab9 mutant, and reduced by the expression of GFP-Rab9^S21N^, a guanosine diphosphate (GDP)-preferring dominant-negative Rab9 mutant (Nishida et al., 2009). Moreover, Rab9 silencing by a targeted siRNA reduced the number of autophagic vacuoles but induced the accumulation of isolation membranes. Numerous isolation membranes are normally generated by etoposide exposure, so siRab9 did not merely slow down the progression of autophagy but rather inhibited autophagosome maturation. The role of Rab9 in the extension and closure of isolation membranes in the alternative autophagy pathway is thought to replace the role of Atg5/Atg7/LC3 in conventional autophagy (Figure 1).

## Physiological role of autophagy in neurodegeneration

Autophagy can be classified into constitutive and inducible types according to the mechanism of induction (Table 1). Whereas constitutive autophagy continuously degrades abnormal proteins to maintain cellular homeostasis, inducible autophagy occurs on a larger scale to suppress cellular injury against various stressors. Autophagy can also be classified into bulk and selective types based on the cargo that is degraded. Autophagy was originally considered to degrade subcellular constituents in a nonselective manner, i.e., bulk autophagy. However, specific molecules and organelles are also digested by selective autophagy during which cargo molecules are recognized by cargo receptors and subsequently enclosed by autophagic structures. According to these classification criteria, the well-studied starvation-induced type of autophagy is considered as inducible and bulk autophagy. Many phenotypes observed in systemic and tissue-specific *Atg* knockout mice, such as defects in preimplantation fetal development (Kuma et al., 2004), are due to the failure of constitutive and selective autophagy. As *Atg7*-deficient mice lack conventional autophagy, they demonstrate neurological defects, including the abnormal limb-clasping reflexes, because of the severe damage to cortical and cerebellar neurons (Komatsu et al., 2006). Furthermore, there was reactive gliosis was observed in association with neuronal loss. Importantly, ubiquitin-positive aggregates were found in neurons. These findings illustrate that conventional autophagy prevents neuronal damage by constitutively eliminating ubiquitin-positive aggregates.

**Table 1:** Classification of autophagy from the view of inducers and substrates.

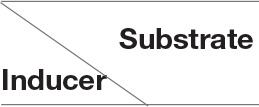	**Bulk autophagy (non-specific degradation)**	**Selective autophagy (specific degradation)**
Constitutive autophagy (maintain homeostasis)	**Cellular metabolism** Replace old proteins/organelles with new ones	**Cell protection** Degrade unfavorable, damaged proteins, and organelles
Inducible autophagy (response to various stimuli)	**Nutrient supply** Starvation-induced Rapamycin-activated	**Cellular adaptation** Cellular stress (DNA damage, etc.) Differentiation

## Neurodegenerative diseases and autophagy in humans

**Figure 2:**
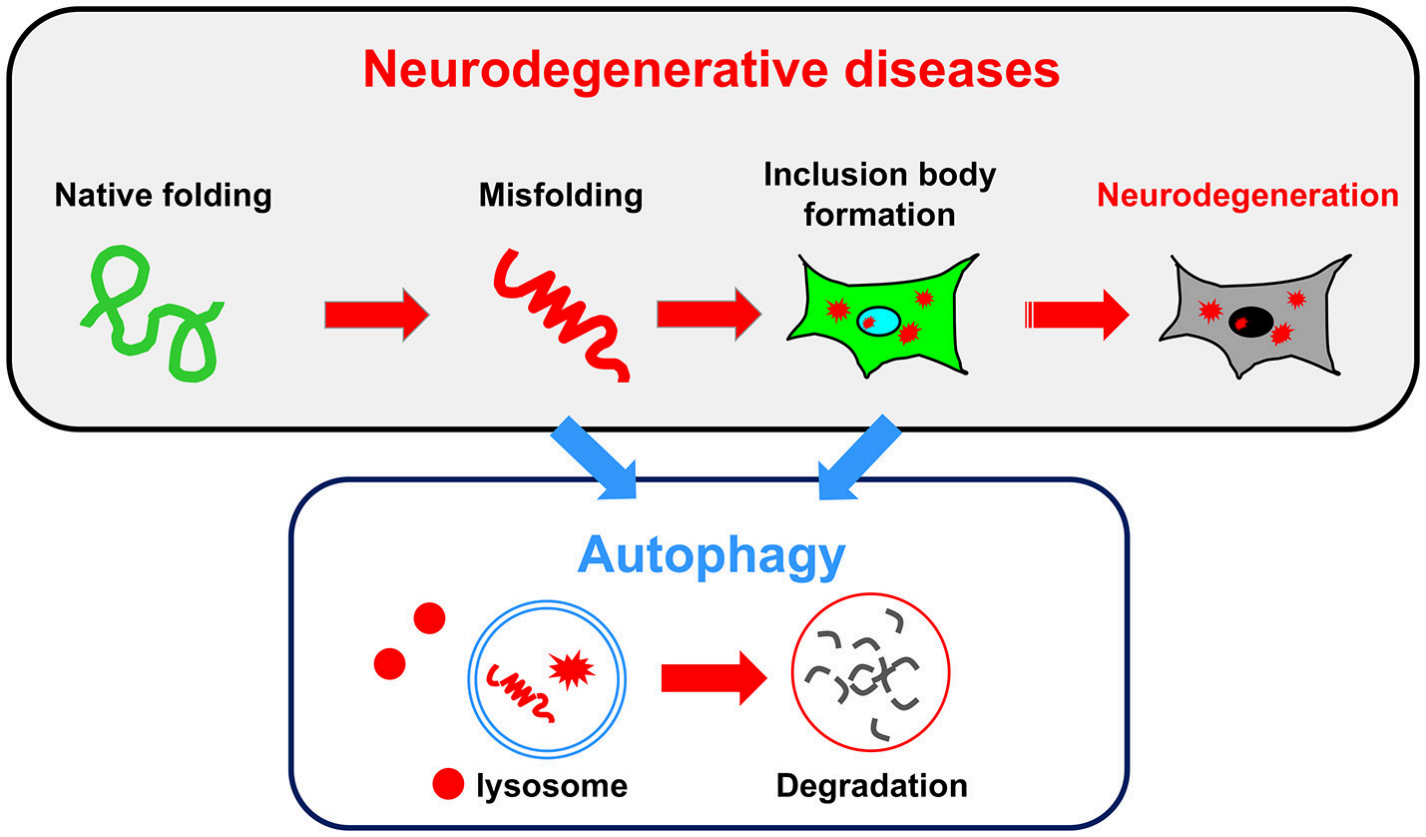
Relationship between neurodegenerative diseases and autophagy. A common pathology shared by several neurodegenerative diseases is the accumulation of misfolded proteins and inclusion bodies. Autophagy may be useful for degrading both misfolded proteins and inclusion bodies.

Consistent with the findings observed in autophagy-deficient mice showing neurodegenerative defects, an impairment of autophagy is often associated with human neurodegenerative diseases. In some cases, autophagy failure is considered as the primary underlying disease pathology. In most neurodegenerative diseases, including ALS, PD, AD, and the polyQ diseases, the accumulation of misfolded proteins is a common pathological hallmark. These misfolded proteins damage neurons, eventually causing their death. Because the reduction of autophagic activity directly affects the accumulation of misfolded proteins, autophagy is a key target pathway for the treatment of neurodegenerative diseases (Figure 2). In this section, we briefly describe the relationship between conventional autophagy and several major neurodegenerative diseases.

## ALS/ TDP-43 proteinopathies

ALS, a fatal neurodegenerative disorder, is primarily a sporadic disease; however, approximately 5–10% of the cases are familial. In particular, mutations in several genes including superoxide dismutase 1 (*SOD1*), TAR DNA-binding protein-43 (*TDP-43*), fused in sarcoma/translocated in liposarcoma (*FUS/TLC*), and/or *C9orf72*, are thought to contribute to the progressive degeneration of motor neurons. A mutation in *SOD1* was the first genetic mutation that was shown to be associated with ALS, and more than 170 *SOD1* mutations have been identified to date. TDP-43, a nuclear factor that controls the fate of cellular RNAs, is found in an aggregated form in neurons of ALS patients, and genetic mutations in *TDP-43* were recently identified in some ALS patients. These mutants commonly fail to fold properly, leading to the accumulation of misfolded proteins in neurons of ALS patients. Numerous studies suggest that autophagy might be a significant regulator of pathological aggregate formation in ALS. For example, the overexpression of transcription factor EB (TFEB), a master regulator of the transcription of autophagy-related genes, led to increases in the expression of Beclin 1 and LC3 and suppressed the cellular toxicity induced by the expression of these mutant proteins (Chen Y. et al., 2015).

Recently, receptors for selective autophagy, such as sequestosome-1 (SQSTM1)/p62 and optineurin (OPTN), as well as TANK-binding kinase 1 (TBK1) were shown to be involved in the degeneration of motor neurons (Maruyama et al., 2010; Teyssou et al., 2013; Freischmidt et al., 2015). Insoluble protein aggregates of these proteins were found to accumulate in motor neurons and surrounding glial cells. Intriguingly, these inclusions were found in the brainstem, spinal cord, cerebellum, hippocampus, and frontal and temporal lobes of ALS patients. SQSTM1 interacts with and sequesters autophagy substrates to isolation membranes via its interaction with LC3, and ALS-causing *SQSTM1* mutants increase the expression levels of TDP-43 (Teyssou et al., 2013). Consistently, mutations in the *SQSTM1* were shown to suppress the recruitment of mutant SOD1 into autophagosomes (Gal et al., 2017). OPTN also plays a role in sequestering cargo to nascent autophagic vacuoles via its interaction with LC3 (Wild et al., 2011). In addition, OPTN interacts with myosin VI for the proper trafficking of autophagosomes (Sahlender et al., 2005). Interestingly, most of the ALS-causing mutations in *OPTN* are located in the myosin VI-binding domain (Sundaramoorthy et al., 2015). These mutants do not interact with myosin VI and are unable to eliminate mutant SOD1 and TDP-43. Finally, TBK1 promotes autophagy by phosphorylating both SQSTM1 and OPTN, and ALS-associated mutations in *TBK1* reduce its binding affinity with OPTN and decrease the clearance of dysfunctional mitochondria (Richter et al., 2016).

Mutations in *C9orf72*, the most common cause of ALS, have been proposed to downregulate autophagy (Ji et al., 2017). C9orf72 is a core component of GDP/ GTP exchange factor (GEF) for Rab8 and Rab39, which is crucial for autophagosome maturation. This molecule also interacts with Ulk1 and advances the autophagy machinery; as such, ALS-associated mutations of *C9orf72* do not interact with Rab proteins, and autophagy fails to proceed (Tang, 2016; Corbier and Sellier, 2017). These findings indicate that selective autophagy, which degrades SOD1 and TDP-43 mutants and requires SQSTM1, OPTN, and TBK1, is crucial for protection from ALS.

Autophagy facilitates the reduction of unfolded proteins. For example, progesterone and lithium were shown to activate autophagic flux, thereby decreasing SOD1 aggregates and improving motor dysfunction in SOD1-mutant mice (Motoi et al., 2014). Consistently, trehalose, a disaccharide that induces mTor-independent autophagy, was demonstrated to inhibit TDP-43 aggregate formation (Wang et al., 2010), whereas the activation of mTor-dependent autophagy by rapamycin also rescued motor dysfunction in TDP-43 transgenic mice (Wang et al., 2012). Not only misfolded protein aggregation but also mitophagy failure contributes to ALS pathology. Disease-associated mutants of OPTN, a key molecule in mitophagy, results in the failure to remove damaged mitochondria, as well as misfolded protein aggregates, and contribute to the neurodegeneration in ALS (Wong and Holzbaur, 2014). Despite the widespread evidence regarding the therapeutic outcomes of autophagy induction in diseased neurons, the role of autophagy is more complex, because n-butylidenephthalide (n-BP), an extract from *Angelica sinensis*, inhibits neuronal autophagy by activating the Akt/mTOR signaling but attenuates neuronal loss and improves motor function (Hsueh et al., 2016). Therefore, autophagy activators do not always show positive effects on ALS progression. The manner in which autophagy is induced is key to its protective or destructive effects in ALS. Studies to further our understanding of the autophagy pathway and its utility as a promising therapeutic strategy for ALS are important.

## Polyglutamine diseases

PolyQ diseases, including Huntington disease (HD), spinal and bulbar muscular atrophy, dentatorubral-pallidoluysian atrophy, spinocerebellar ataxia (SCA) types 1, 2, 3, 6, 7, and 17, and HD-like 2 are inherited progressive neurodegenerative diseases elicited by expansions of CAG triplet repeats within specific genes. PolyQ tracts in mutant proteins, i.e., mutant Huntingtin (Htt) in HD, encoded by the CAG repeat, are responsible for the formation of toxic oligomers and aggregates. The length of the polyQ tract is crucial, because longer tracts are more prone to form aggregates. These mutant proteins accumulate and form intracellular inclusions, and previous studies showed that autophagy degrades not only these inclusions but also soluble mutant proteins. Importantly, the degradation of mutant proteins was shown to decrease neuronal damage and improve neurodegenerative deficits in mouse models of the polyQ diseases.

Recent studies have increasingly focused on the perturbation of autophagy in the polyQ diseases, such as that observed in striatal neurons in HD mouse models and lymphocytes in HD patients. In HD cells, cargo recognition was shown to be significantly impaired due to the failure of p62 interaction with mutant Htt, which leads to the inefficient selective degradation of mutant Htt (Fu et al., 2017). Moreover, because the accumulated mutant Htt can potentially trap and inactivate Beclin 1 (Shibata et al., 2006), autophagy is significantly suppressed at the autophagosome-lysosome fusion stage. Mutant Htt also interacts with and inactivates Ras homolog enriched in striatum (Rhes), an autophagy regulator (Mealer et al., 2014). Rhes accelerates autophagy by inhibition of the interaction of Beclin 1 with Bcl-2. The inhibition of autophagy leads to the further accumulation of mutant Htt and neuronal injury. Consistently, in SCA7-mutant patients and mice, the accumulation of p62 and ubiquitin were observed, indicating the impairement of autophagic activity (Alves et al., 2014). Furthermore, the androgen receptor with an expanded polyQ repeat was shown to interact and attenuate the function of TFEB (Song et al., 2013; Cortes et al., 2014). Although studies suggesting that autophagic activity is altered in mouse models of polyQ disease are controversial, an impairment of autophagy is expected to occur in many of the neurons with polyQ accumulation.

PolyQ disease pathogenesis is strongly affected by neuronal autophagic activity. Therefore, the activation of autophagy can potentially prevent the accumulation of aggregate-prone proteins, thus inhibiting or slowing the progression of neurodegeneration. Upregulating autophagy with rapamycin reduced the formation of aggregates and cytotoxicity in cells *in vitro* and in a fly model of HD *in vivo* (Ravikumar et al., 2002, 2004). CCI-779, a rapamycin analog, also improved behavioral and motor performance in mouse models of HD and SCA3 (Ravikumar et al., 2004). Other autophagy inducers, such as berberine, metformin, rilmenidine, and trehalose also activate autophagy, decrease polyQ aggregation, and improve behavioral performance in polyQ disease mouse models (Tanaka et al., 2004; Ma et al., 2007; Rose et al., 2010; Chen Z. Z. et al., 2015; Jiang et al., 2015). Metformin is an antidiabetic drug that activates AMPK-dependent autophagy (Poels et al., 2009), and rilmenidine is used for hypertension and activates mTor-independent autophagy (Rose et al., 2010). Both drugs are already used clinically in humans. In contrast, in a mouse model of SCA3, the combination of CCI-779 with LiCl showed no beneficial effects, but rather toxic effects (Duarte-Silva et al., 2016). Clinical trials have been performed to assess their utility in neurodegenerative diseases.

## Alzheimer disease

AD is a chronic neurodegenerative disease that causes memory and cognitive deficits. AD is characterized by the accumulation of neurotoxic extracellular beta-amyloid (Aβ) plaques and hyperphosphorylated tau in intracellular neurofibrillary tangles (NFTs) in the brain. Aβ is generated from amyloid precursor protein (APP) by two cleavage events. Aβ homeostasis in the brain is believed to play a key role in the progression of neurodegeneration, and several studies demonstrated that various clearance mechanisms play pivotal roles in the development of AD. However, the role of autophagy in AD pathogenesis remains controversial. Numerous studies have reported the degradation of Aβ and APP by autophagy and the reduction of Aβ expression by the upregulation of autophagy, similar to that observed with other proteins associated with neurodegenerative diseases. However, some studies have reported the requirement of autophagy in AD pathogenesis. These contradictory findings stem from studies showing that Aβ generation was suppressed following the inhibition of autophagy by 3-methyl adenine (Zheng et al., 2011). Furthermore, this idea is supported by the presence of APP and Aβ in purified autophagic vacuoles from brains of AD patients. In these vacuoles, presenilin-1 (PS-1), an enzyme that cleaves APP, is highly enriched. Autophagy was also shown to contribute to Aβ secretion, a crucial event in AD pathogenesis. Delivery of Aβ to the extracellular space and plaque formation were significantly reduced due to the lack of Atg7 in APP transgenic mice, and restoration of autophagy restored Aβ secretion to normal levels (Nilsson et al., 2013).

The downregulation of autophagy was morphologically and genetically demonstrated in AD brains. Morphologically, numerous immature autophagic vacuoles were observed in dystrophic neurites of AD patients, suggesting the impairment of trafficking of autophagic vacuoles or autophagosome-lysosome fusion (Nixon et al., 2005). Genetically, Beclin 1, a major player in autophagy, was identified as a causative molecule in AD pathology. AD brains were reported to have decreased expression levels of Beclin 1 early in the disease process (Pickford et al., 2008). A reduction in Beclin 1 levels was shown to attenuate autophagic activity, which likely facilitates the proteolytic cleavage of APP. Another factor that was shown to be associated with AD is phosphatidylinositol-binding clathrin assembly protein (PICALM), which plays a role in autophagy (Tian et al., 2013) by the trafficking of soluble NSF attachment protein receptors. PICALM was also reported to play a role as an autophagy receptor that sequesters APP to LC3-positive autophagosomes (Tian et al., 2014). Genetic studies showing a reduction in PICALM indicate that not only bulk autophagy but also selective autophagy for APP might be suppressed in AD brains. Mutations in PS-1 cause familial AD via the alteration of APP processing. However, recently, another pathological role for mutant PS-1 was proposed, which includes inhibition of the maturation of the lysosomal v-ATPase that leads to an increase in lysosomal pH and a consequent reduction in autophagic cargo elimination (Lee et al., 2010). This process might be mediated by the failure of the ER chaperone function of PS-1, which facilitates the proper assembly of the V_0_a1 subunit of the v-ATPase.

Whereas the role of autophagy in Aβ generation remains controversial, various autophagy-related compounds were tested for potential therapeutic utility. Lithium and berberine, which are both autophagy inducers targeting the Ulk1-pathway, were demonstrated to induce autophagy and exert neuroprotective effects through the regulation of APP processing and a reduction in Aβ levels in the CRND8 transgenic mouse model of AD (Fiorentini et al., 2010; Durairajan et al., 2012). Both trehalose and carbamazepine were shown to exert protective effects on AD mice via mTor-independent autophagy (Du et al., 2013; Li et al., 2013). Carbamazepine was shown to significantly enhance autophagic flux in the APP(swe)/PS-1(deltaE9) AD mouse model and reduce the cerebral amyloid plaque burden and Aβ 1–42 levels (Li et al., 2013). Latrepirdine stimulates Atg5-dependent autophagy and reduces the accumulation of toxic Aß aggregates in AD mice (Steele et al., 2013). Rapamycin and SMER28, a small-molecule rapamycin enhancer, both activate autophagy and decrease the expression levels of Aß 1–42 (Spilman et al., 2010; Tian et al., 2011), which is the most amyloidogenic form of Aß, in PD-APP transgenic mice, one of the earliest mouse models of AD. Taken together, these findings suggest that, although the exact pathological role of autophagy in AD remains to be elucidated, autophagy inducers might provide a new effective therapeutic strategy by degrading aggregates in the early stages of AD. In contrast, the activation of autophagy might enhance disease severity during the late stages of AD, by accelerating Aβ production.

## Tauopathies

Tau is a microtubule-binding protein that attaches to and stabilizes microtubules. Tauopathies are a constellation of neurodegeneration diseases, including AD, frontotemporal lobar degeneration (FTLD), progressive supranuclear palsy (PSP), and corticobasal degeneration (CBD), in which hyperphosphorylated tau accumulates in intracellular tangles and extracellular Aβ deposits. The phosphorylation of tau by glycogen synthase kinase 3β facilitates its accumulation within the cytosol (Sperber et al., 1995). Similar to that observed with other proteins implicated in neurodegenerative diseases, the inhibition of autophagy, such as that activated by chloroquine, causes a delay in tau clearance and aggregation (Hamano et al., 2008). Furthermore, phosphorylated tau accumulation is higher in brain-specific *Atg7* knockout mice than control mice (Inoue et al., 2012), indicating that phosphorylated tau is a substrate of conventional autophagy. Consistently, phosphorylated tau colocalizes with LC3 and p62 proteins in postmortem brain specimens from patients with AD, PSP, and CBD, likely because these proteins are engulfed into autophagosomes but are not degraded by autolysosomes (Piras et al., 2016). These studies suggest that an impairment of autophagy plays a crucial role in the progression of tauopathies, and that autophagy facilitates the degradation of phosphorylated tau to maintain it at low levels.

Based on these findings, the induction of autolysosome formation appears to be a good therapeutic strategy for the tauopathies. Indeed, the activation of autophagy using rapamycin or temsirolimus, an analog of rapamycin, was shown to alleviate tau phosphorylation in neurons and to improve the progression of tau pathology in P301S mutant human tau transgenic mice (Ozcelik et al., 2013; Jiang et al., 2014). Furthermore, trehalose, and methylthioninium chloride were shown to reduce tau aggregates and improve neuronal survival in several mouse models of the tauopathies (Congdon et al., 2012; Schaeffer et al., 2012). Finally, metformin, which inhibits tau hyperphosphorylation via the inhibition of TORC1, is currently being tested for its utility against AD in several clinical trials (Kickstein et al., 2010).

## Parkinson disease

PD is the second most common neurodegenerative disease following AD. The main underlying pathology of PD is functional failure and subsequent death of dopaminergic neurons in the substantia nigra, which leads to muscle rigidity, bradykinesia, resting tremor, and even neurocognitive impairment. Many of the dopaminergic neurons in PD patients contain round, cytoplasmic inclusions called Lewy bodies that are comprised of α-synuclein and ubiquitin-positive proteins. Most PD cases are sporadic, and 5–10% are familial, due to mutations in various PD-associated genes that encode for PD-associated proteins (PARK).

One subtype of familial PD arises from the A53T mutation in α-synuclein, which is encoded by *PARK1*. The level of α-synuclein in neurons is a key to neurotoxicity in PD; therefore, efficient α-synuclein degradation is a crucial determinant of PD severity. Findings from recent studies indicate that deubiquitinated α-synuclein is the main target of autophagy (Rott et al., 2011), and that degradation of stress-increased, rather than basal α-synuclein was crucial in a transgenic mouse model (Wu et al., 2016). In contrast, inclusions containing α-synuclein were shown to reduce autophagic activity during the maturation of autophagosomes and their fusion with lysosomes (Button et al., 2017). Furthermore, α-synuclein itself was also reported to suppress autophagy by the induction of Atg9 mislocalization, which was observed in α-synuclein transgenic mice (Winslow et al., 2010). Interestingly, similar Atg9 mislocalization was observed in patients with the D620N mutation of vacuolar protein sorting-associated protein 35 (Vps35; PARK17) (Zavodszky et al., 2014). Atg9 is a Golgi-localizing protein, whereas Vps35 regulates various Golgi proteins by affecting the retromer complex. Therefore, the Vps35 mutant fails to appropriately localize Atg9 and leads to impaired autophagy.

Leucine-rich repeat serine-threonine protein kinase-2 (*LRRK2*; *PARK8*) is another PD-causing gene. LRRK2 colocalizes with Lewy bodies in some PD patient brains. LRRK2 is a large protein containing a Ras-like small GTPase domain and a MAPKKK-like kinase domain. Loss of LRRK2 causes the accumulation of α-synuclein phosphorylated on Ser129 and the impairment of protein degradation pathways, leading to apoptotic cell death in aged mice (Tong et al., 2010). The G2019S LRRK2 mutation is found not only in familial PD patients but also in 1–2% of patients with sporadic PD (Goldwurm et al., 2005). LRRK2 was suggested to suppress autophagy, because silencing of *LRRK2* or inhibition of its kinase activity was shown to increase autophagic flux (Alegre-Abarrategui et al., 2009).

Loss-of-function mutations in the P-type ATPase ATP13A2 (PARK9) causes early-onset PD (Ramirez et al., 2006). Because lysosomal ATPase is essential for the maintenance of lysosomal pH and autophagosome-lysosome fusion, mutations in ATP13A2 impair these processes, resulting in the accumulation of autophagosomes. A defect in autophagy accelerates the accumulation of α-synuclein, thereby worsening the disease.

As described above, autophagy contributes to the suppression of various neurodegenerative processes by degrading unfolded proteins. In addition, autophagy functions to inhibit certain PD types by degrading damaged mitochondria. Among the genes associated with familial PD, Parkin (*PARK2*) and Pink1 (*PARK6*) eliminate damaged mitochondria by autophagy (Clark et al., 2006; Park et al., 2006). This mechanism, termed mitophagy, starts with the accumulation of Pink1 on the outer mitochondrial membrane from the cytosol following mitochondrial depolarization. Next, Parkin, a cytosolic E3-like ligase, associates with Pink1 on the outer mitochondrial membrane, which leads to ubiquitination of depolarized mitochondria by the ubiquitin ligase activity of Parkin and recruitment of several autophagy components, including p62 (Geisler et al., 2010). Finally, ubiquitinated mitochondria are recognized by autophagic molecules and are digested by autophagy. Thus, genetic mutations in Parkin and Pink1, which cause early-onset familial and sporadic PD, fail to eliminate damaged mitochondria, which culminates in the degeneration of dopaminergic neurons. Importantly, Parkin-mediated mitophagy is significantly suppressed by the lack of Atg5 (Huang et al., 2011).

Based on these data, reduction of α-synuclein aggregation and the induction of mitophagy by autophagy might be useful therapeutic approaches against PD. In cell-based assays, several polyphenols, including resveratrol demonstrated autophagy-inducing activity and might be useful as therapeutic agents for PD. Kaempferol and celastrol also prevent rotenone-induced SH-SY5Y cell loss via autophagy activation (Filomeni et al., 2012; Deng et al., 2013). Furthermore, microencapsulated rapamycin improved motor function in mice overexpressing α-synuclein (Bai et al., 2015). Oral administration of trehalose also activated autophagy and suppressed insoluble α-synuclein accumulation (Tanji et al., 2015), whereas nilotinib, a receptor tyrosine kinase inhibitor approved for chronic myelogenous leukemia, was also shown to inhibit the protein phosphatase PP2A and induce AMPK phosphorylation and autophagy, resulting in modulation of the neuroimmune response to α-synuclein expression in mice (Yu et al., 2013).

## Closing remark

In this review, we described two distinct autophagic pathways, namely, conventional and alternative autophagy, and compared their characteristics. We also described the involvement of conventional autophagy in the progression of many neurodegenerative diseases. However, the association between alternative autophagy and neurodegenerative diseases has not been fully clarified to date. In this review, we also summarized the potential uses of conventional autophagy for the treatment of various neurodegenerative diseases. Given that the induction of autophagy can potentially mitigate disease severity, various autophagy-inducing compounds have been developed and evaluated in various rodent models of the neurodegenerative diseases. However, most compounds have multiple functions, and hence it is difficult to confirm that their therapeutic effects are a result of autophagy. Detailed analysis using rodent models are expected to clarify the effect of autophagy-inducing therapies on neurodegenerative diseases.

## Author contributions

NF, MS contributed to the design of this review. SS described the manuscript.

### Conflict of interest statement

The authors declare that the research was conducted in the absence of any commercial or financial relationships that could be construed as a potential conflict of interest.
